# Microbial production of Propionic and Succinic acid from Sorbitol using *Propionibacterium acidipropionici*

**DOI:** 10.1186/s13568-015-0095-6

**Published:** 2015-02-20

**Authors:** Juliana C Duarte, Gustavo P Valença, Paulo J S Moran, J Augusto R Rodrigues

**Affiliations:** Institute of Chemistry, University of Campinas, Campinas, SP 13083-970 Brazil; School of Chemical Engineering, University of Campinas, Campinas, SP 13083-852 Brazil

**Keywords:** *Propionibacterium acidipropionici*, Sorbitol, Propionic acid, Succinic acid, Batch fermentation

## Abstract

Three sequential fermentative batches were carried out with cell recycle in four simultaneously operating bioreactors maintained at pH 6.5, 30°C, and 100 rpm. *P. acidipropionici* ATCC 4875 was able to produce propionic and succinic acid from sorbitol. The concentration of propionic acid decreased slightly from 39.5 ± 5.2 g L^−1^ to 34.4 ± 1.9 g L^−1^, and that of succinic acid increased significantly from 6.1 ± 2.1 g L^−1^ to 14.8 ± 0.9 g L^−1^ through the sequential batches. In addition, a small amount of acetic acid was produced that decreased from 3.3 ± 0.4 g L^−1^ to 2.0 ± 0.3 g L^−1^ through the batches. The major yield for propionic acid was 0.613 g g^−1^ in the first batch and succinic acid it was 0.212 g g^−1^ in the third batch. The minor yield of acetic acid was 0.029 g g^−1^, in the second and third batches.

## Introduction

*Propionibacterium acidipropionici* has been widely studied for the heterofermentative production of propionic acid, including fermentation on a semi-industrial scale (Zhu et al. 2010). Propionic acid and its salts are valuable industrial products with several applications such as mold-inhibitors, preservatives for animal and human food, fruit flavorings, additives in cellulosic plastics, and herbicides and medications for animal therapy (Boyaval and Corre 1995). Consumption by the animal world was estimated at 293.4 thousand tons in 2009, representing a market of approximately $530 million with an expected rate of 3.9% until 2014 (Bizzari and Gubler 2004). Currently, industrial production of propionic acid utilizes fossil-based resources. However, the finite nature of oil and the rise in its price increased customer awareness and demand for green products. Furthermore, increased costs waste disposal and restrictions on land filling for certain types of waste led to increased interest in a more sustainable production of chemicals and materials from renewable bio-based raw materials (Tsoskounogiou *et al.*^2008^). The conversion of bio-based residues or by-products into valuable chemicals offers several potential advantages: low product cost, less environmental impact, less energy requirement, and less toxic products.

The production of propionic acid by fermentation using *Propionibacterium sp* has been investigated during the last decade*.* These microorganisms are able to grow and produce propionic acid using several cheap industrial and agricultural by-products and residues that serve as C-source. These cheap sources are biodiesel glycerol (Ruhal *et al.*^2011^), molasses (Feng *et al.*^2011^), fish hydrolysate (Mahmoud and Levin 1993), lactose whey (Yang *et al.*^1995^), hydrolyzed corn meal (Huang et al. 2002), glucose (Koussémon *et al.*^2003^), wheat flour (Kagliwal *et al.*^2013^; Sabra *et al.*^2013^), and several other by-products including mixtures of glycerol and glucose (Wang and Yang 2013).

Succinic acid has a wide range of industrial application, for example as a chemical intermediate for the production of lacquers and perfume esters as well as flavoring, bacteriostatic, or neutralizing agent in the food industry. Furthermore, succinic acid also has a special chemical market for the production of coatings, surfactants dyes, detergents, green solvents, biodegradable plastics, and the stimulation of animal and plant growth. With its linear and saturated dicarboxylic acid structure, succinic acid can be readily converted to other chemicals, such as 1,4-butanediol (Minh *et al.*^2010^), gamma-butyrolactone, tetrahydrofuran, adipic acid, *N*-methylpyrrolidone, or linear aliphatic esters. With various environmental implications, the demand for succinic acid is expected to increase significantly. A new biodegradable polymer, poly (1,3-propylene succinate), can be derived by the polycondensation of succinic acid with 1,3-propanediol and also with thermoplastic poly(butylene succinate) (Ranucci *et al.*^2000^). While the current global succinic acid production is approximately 30,000 to 50,000 tons per year with a market price of US$ 2400–3000 per ton, the market is expected to reach 100,000 tons per year by 2015 (Adsul *et al.*^2011^).

Succinic acid can be produced via chemical routes by paraffin oxidation, catalytic hydrogenation, or electroreduction of maleic acid or maleic anhydride (Muzumdar *et al.*^2004^). Recent developments have focused on biotechnological alternatives, in particular microbial transformation based on the use of renewable biomass as feedstock (Cheng *et al.*^2012^; Hatti-Kaul *et al.*^2007^).

Notably, even after 100 years of accumulated research on *propionibacteria*, propionic acid is still produced via petrochemical routes, and no industrial biotechnological process has been established for these organisms. The main hindrances have been low productivity, low final product concentration, slow growth, high end-product inhibition, and costly downstream separation from sub-products (Blanc and Goma 1987; Goswami and Srivastava 2001). Our interest in the present article is to explore new sources of materials and particularly sorbitol. This inexpensive polyol has been subjected to fermentation by *P. acidipropionici* in a PhD thesis (Suwannakham 2005), but until now no article has been published. Sorbitol has a high reduction degree (4.33) (VanBriesen 2002) that favors the production of more reduced metabolites. We expect to minimize the amount of acetic acid produced and to maximize the formation of propionic and succinic acid in a fermentation process using *P. acidipropionici* ATCC 4875.

## Materials and Methods

### Chemicals

Sorbitol was purchased from Sigma-Aldrich Co., USA, and yeast extract from Oxoid Ltd., England. CaCl_2_.2H_2_O, CoCl_2_.6H_2_O, MnSO_4_.H_2_O, ZnSO_4_.7H_2_O, KH_2_PO_4_, and (NH_4_)_2_HPO_4_ were purchased from Synth Ltda., Brazil. MgSO_4_.7H_2_O was purchased from Nuclear, Brazil and FeSO_4_.7H_2_O was purchased from Vetec Ltda, Brazil.

### Bioreactors

A 3.6 L Infors-HT-Labors bioreactor was used for biomass growth, and two 0.5 L Infors-HT-Multifors, each one equipped with two parallel vessels, were used to promote batch fermentations. All bioreactors are equipped with pH and temperature sensors, agitation, and N_2_ flow control.

### Microorganism’s growth and fermentation medium

The *Propionibacterium acidipropionici* ATCC 4875 used in this study was grown in a synthetic medium using 10 g L^−1^ sorbitol as a carbon source, 10 g L^−1^ yeast extract, 1 g L^−1^ KH_2_PO_4_, 2 g L^−1^ (NH_4_)_2_HPO_4_, and the following micronutrients: 5 mg L^−1^ FeSO_4_.7H_2_O, 10 mg L^−1^ MgSO_4_.7H_2_O, 2.5 mg L^−1^ MnSO_4_.H_2_O, 5 mg L^−1^ ZnSO_4_.7H_2_O, 10 mg L^−1^ CaCl_2_.2H_2_O, 10 mg L^−1^ CoCl_2_.6H_2_O (Coral *et al.*^2008^). In the fermentation medium, only the sorbitol concentration was changed from 10 to 80 g L^−1^. The dry cell weight (DCW) was calculated from the OD_600_ value. One unit of OD_600_ was equivalent to 0.431 g L^−1^ DCW. The meaning of these abbreviations can be found in Table 1.

**Table 1:** **List of nomenclature**

**Abbreviations**	**Meaning**
**Y**_**X/S**_	Yield of biomass with respect to substrate (g g^−1^)
**Y**_**SA/S**_	Yield of succinic acid with respect to substrate (g g^−1^)
**Y**_**AA/S**_	Yield of acetic acid with respect to substrate (g g^−1^)
**Y**_**PA/S**_	Yield of propionic acid with respect to substrate (g g^−1^)
**Y**_**SA/X**_	Yield of succinic acid with respect to biomass (g g^−1^)
**Y**_**AA/X**_	Yield of acetic acid with respect to biomass (g g^−1^)
**Y**_**PA/X**_	Yield of propionic acid with respect to biomass (g g^−1^)
**m**	Cell maintenance (g g^−1^ h^−1^)
**r**_**s**_	Instantaneous substrate consumption rate (g h^−1^)
**r**_**SA**_	Instantaneous succinic acid production rate (g h^−1^)
**r**_**AA**_	Instantaneous acetic acid production rate (g h^−1^)
**r**_**PA**_	Instantaneous propionic acid production rate (g h^−1^)
**r**_**x**_	Instantaneous cell growth rate (g h^−1^)
**μ**_**s**_	Specific substrate consumption rate (h^−1^)
**μ**_**SA**_	Specific succinic acid production rate (h^−1^)
**μ**_**AA**_	Specific acetic acid production rate (h^−1^)
**μ**_**PA**_	Specific propionic acid production rate (h^−1^)
**μ**_**x**_	Specific cell growth rate (h^−1^)
**DCW**	Dry cell weight
**OD**_**600**_	Optical density at 600 nm
**P/A**	Ratio of propionic to acetic acid (mol mol^−1^ or g g^−1^)
**S/A**	Ratio of succinic to acetic acid (mol mol^−1^ or g g^−1^)
**FBB**	Fibrous bed bioreactor

### Pre-inoculum and inoculum preparation

The pre-inoculum was prepared using 1.5 mL of an ultra-low temperature preserved culture that was thawed on ice and transferred to a 15 mL screw-cap flask containing 13.5 mL of the growth medium described above and then incubated at 30°C for 24 h without agitation. After 24 h the inoculum was prepared by transferring 5 mL of the pre-inoculum to a 50 mL screw-cap flask containing 45 mL of the growth medium described above (in duplicate). *P. acidipropionici* was incubated at 30°C for 48 – 50 h without agitation (final OD_600_ ~ 2.5), and the total inoculum amount (100 mL) was inoculated into 900 mL of the fermentation medium in a Infors-HT Labfors bioreactor to promote biomass growth.

### Biomass growth

To promote biomass growth, *P. acidipropionici* was grown in 1 L of the fermentation medium described above (sorbitol 80 g L^−1^). Growth was carried out in the Infors-HT Labfors bioreactor for 48 h at 30°C, pH 6.5 (NaOH 4 mol L^−1^), and 100 rpm under anaerobic conditions (N_2_ bubbling) for the first 30 minutes. The medium was then divided in four 500 mL screw-cap flasks, each one containing 250 mL of medium, and centrifuged at 3000 rpm for 20 minutes. All the supernatants were discarded and the cells were suspended in 250 mL of fresh fermentation medium.

### Sequential batch fermentation

Sequential batch fermentations were performed in two independent Infors-HT Multifors bioreactors, each one equipped with two parallel vessels operating simultaneously and containing 250 mL of fresh fermentation medium as described above. Each sequential batch fermentation was carried out for 70 h at 30°C, pH 6.5 (NaOH 4 mol L^−1^), and 100 rpm under anaerobic conditions (N_2_ bubbling) for the first 15 minutes. Samples of 1.5 mL were aseptically removed at the beginning of the fermentation and at periodic intervals of 24 h. After 70 h, each medium was transferred to a 500 mL screw-cap flask and centrifuged at 3000 rpm for 20 minutes. The supernatants were discarded and the cells were suspended in a fresh medium. The flasks containing the cells in fresh medium were transferred aseptically to the bioreactor to start new batch fermentation. Each batch was simultaneously made in quadruplicate.

### Quantitative analysis

#### Carbon source and organic acids determination

Sorbitol, succinic, acetic, and propionic acid concentrations were determined by high-performance liquid chromatography (Agilent 1200 series) using an Aminex® HPX-87H ion exclusion column (Bio-Rad, Hercules, CA, USA) operated at 50°C, with 5 mmol L^−1^ H_2_SO_4_ as the mobile phase at 0.6 mL min^−1^ flow rate. Refraction Index was used as detector. Samples for analysis were centrifuged at 5000 rpm for 15 minutes, filtered through 0.22-μm pore-size filters (Millipore), and diluted with purified water (milliQ). The product and substrate concentrations were calculated using the peak areas in calibration curves equations.

## Results

**Figure 1:**
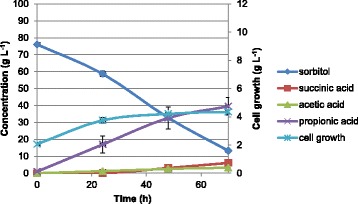
**Profile of sorbitol fermentation by** ***P. acidipropionici*** **for the first batch operating in four independent vessels simultaneously.**

**Figure 2:**
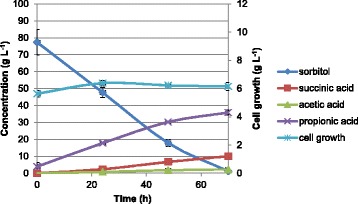
**Profile of sorbitol fermentation by** ***P. acidipropionici*** **for the second sequential batch operating in four independent vessels simultaneously.**

**Figure 3:**
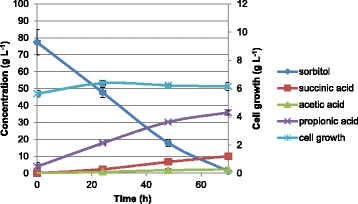
**Profile of sorbitol fermentation by** ***P. acidipropionici*** **for the third sequential batch operating in four independent vessels simultaneously.**

**Figure 4:**
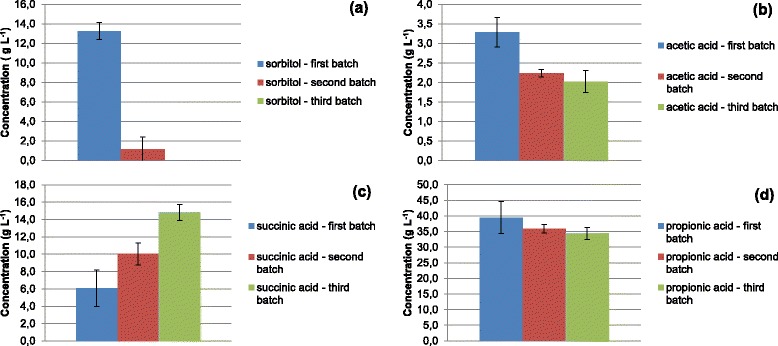
**Sorbitol and organic acids concentration at the end of each sequential batch (each batch was carried out in four independent vessels simultaneously).** **a)** Sorbitol, **b)** Acetic acid, **c)** Succinic acid and **d)** Propionic acid.

Three sequential batches in two independent bioreactors, each one with two parallel vessels (four reactors) operating simultaneously, were carried out with *P. acidipropionici* ATCC 4875 using sorbitol as a carbon source. The results for the first batch are presented in Figure 1. It was observed that sorbitol was not completely consumed after 70 h, and its final concentration was 13.3 ± 0.9 g L^−1^. The final concentrations of succinic, acetic, and propionic acid were 6.1 ± 2.1, 3.3 ± 0.4, and 39.5 ± 5.2 g L^−1^, respectively. Cell growth was also observed, and the final cellular concentration was 4.3 ± 0.1 g L^−1^. In the second sequential batch, contrarily to the first batch, the substrate consumption was almost complete after 70 h, and its final concentration was 1.2 ± 1.3 g L^−1^. The final concentrations of succinic, acetic, and propionic acid were 10.0 ± 1.3, 2.2 ± 0.1, and 35.8 ± 1.4 g L^−1^, respectively. The final concentration of succinic acid increased while that of acetic and propionic acid decreased, compared with the first batch. As expected, cell growth was observed, and the final cellular concentration increased up to 6.1 ± 0.3 g L^−1^ (Figure 2). In the last sequential batch, substrate consumption was completed before 70 h, and the final concentrations of succinic, acetic, and propionic acid were 14.8 ± 0.9, 2.0 ± 0.3, and 34.4 ± 1.9 g L^−1^, respectively. Cell growth was still observed, and the final cellular concentration increased to 9.9 ± 0.5 g L^−1^ (Figure 3). Accordingly, the final concentration of succinic acid increased (from 6.1 ± 2.1 g L^−1^ in the first batch to 14.8 ± 0.9 g L^−1^ in the third sequential batch), that of acetic acid decreased (from 3.3 ± 0.4 g L^−1^ in the first batch to 2.0 ± 0.3 g L^−1^ in the third sequential batch) as well as that of propionic acid (from 39.5 ± 5.2 g L^−1^ in the first batch to 34.4 ± 1.9 g L^−1^ in the third sequential batch) in the sequential batches. A profile change was noticed in sorbitol consumption and product formation throughout the sequential batches, as shown in Figure 4. Furthermore, the overall final results and the carbon recovery for each sequential batch are summarized in Table 2.

**Table 2:** **Substrate, organic acids, cellular final concentrations, and recovered carbon for the three sequential fermentation batches using** ***P. acidipropionici*** **operating in four independent vessels**

	**First batch**	**Second batch**	**Third batch**
Sorbitol (g L^−1^)	13.3 ± 0.9	1.2 ± 1.3	0.0 ± 0.0
Succinic acid (g L^−1^)	6.1 ± 2.1	10.0 ± 1.3	14.8 ± 0.9
Acetic acid (g L^−1^)	3.3 ± 0.4	2.2 ± 0.1	2.0 ± 0.3
Propionic acid (g L^−1^)	39.5 ± 5.2	35.8 ± 1.4	34.4 ± 1.9
Cell (g L^−1^)	4.3 ± 0.1	6.1 ± 0.3	9.9 ± 0.5
Carbon recovery (%)	89.2 ± 5.3	72.3 ± 3.9	74.5 ± 4.2

## Discussion

In the literature, glycerol has been extensively employed as a carbon source in propionic acid fermentations by *P. acidipropionici*. Dishisha et al. (2013), employed glycerol and potato juice in a fermentative process using high-cell-density sequential batches with cell recycle. In their study, final propionic acid concentrations of 43.8 and 50.8 g L^−1^ were reached using glycerol and potato juice, respectively. In the literature, another study (Liu *et al.*^2011^) reports batch processes also using glycerol as a carbon source. In this study, the final concentrations of propionic, acetic, and succinic acid reached 18.1 ± 0.6, 0.54 ± 0.09, and 1.10 ± 0.05 g L^−1^, respectively. In this same study, Liu et al. also report results using glucose as a carbon source; the final concentrations of propionic, acetic, and succinic acid reached 11.5 ± 0.45, 2.57 ± 0.12, and 0.55 ± 0.03 g L^−1^, respectively. According to the results in the literature, the final concentration of propionic acid was higher when using glycerol and potato juice as a carbon sources in a high-cell-density process with sequential batches and cell recycle than that obtained in our studies using sorbitol. However, when using glycerol in batch processes, the final concentrations of propionic and succinic acid were lower than those obtained using sorbitol as a carbon source, as in the present work. In addition, fermentations employing sorbitol as a carbon source produced the most interesting results when compared to those obtained with glucose.

The percentages of recovered carbon were 89.2 ± 5.3, 72.3 ± 3.9, and 74.5 ± 4.2% in the first, second, and third sequential batches, respectively. These results can be explained by the following hypothesis: the cells only used approximately 10% of the carbon source for maintenance, and the cell growth in the first batch and in the second and third sequential batches used approximately 30% due to the increase in cell concentration.

In sorbitol fermentations, a cellular maintenance coefficient of 0.039 g g^−1^ h^−1^ was obtained in the first batch, which increased from 0.044 g g^−1^ h^−1^ to 0.051 g g^−1^ h^−1^ in the second and third sequential batches. (Table 3 – The meaning of abbreviations in Table 3 can be found in Table 1). These results are similar to those obtained by Goswami and Srivastava (2000) using lactose (initial concentration of 47.7 g L^−1^) as the carbon source in a fed-batch experiment, where the cellular maintenance coefficient was 0.038 g g^−1^ h^−1^. Table 3, shows acetic acid yields, Y_AA/S_, of 0.029 g g^−1^ for the second and third batches. These results are similar to those obtained by Zhang and Yang (2009), using an adapted culture of *P. acidipropionici* in FBB fermentation (0.027 ± 0.003 g g^−1^). However, Blanc and Goma (1987), obtained a 0.140 g g^−1^ yield for acetic acid. Accordingly, when compared with household refuse enzymatic hydrolysate as a carbon source, sorbitol fermentation showed lower values of acetic acid yield. The most interesting result obtained for succinic acid yield, Y_SA/S_ in Table 3, is for the third batch (0.212 g g^−1^). Zhang and Yang (2009) obtained lower results for Y_SA/S_ (0.073 ± 0.002 g g^−1^). In the present study, Y_X/S_ was 0.366 g g^−1^ in the first batch, similar to the 0.362 g g^−1^ value obtained by Goswami and Srivastava (2000) in their study with lactose as a carbon source. The definitions of the abbreviations used in Table 3 can be found in Table 1.

**Table 3:** **Yield coefficients and cellular maintenance (m) for the three sequential sorbitol fermentation batches using** ***P. acidipropionici*** **operating in four independent vessels**

	**First batch**	**Second batch**	**Third batch**
Y_X/S_ (g g^−1^)	0.366	0.326	0.279
Y_SA/S_ (g g^−1^)	0.097	0.132	0.212
Y_AA/S_ (g g^−1^)	0.052	0.029	0.029
Y_PA/S_ (g g^−1^)	0.613	0.419	0.438
Y_SA/X_ (g g^−1^)	0.264	0.404	0.760
Y_AA/X_ (g g^−1^)	0.143	0.090	0.102
Y_PA/X_ (g g^−1^)	1.673	1.288	1.570
m (g g^−1^ h^−1^)	0.039	0.044	0.051

In our study, the productivity of propionic acid was approximately 0.5 g L^−1^ h^−1^ (Table 4). (Blanc and Goma 1987) obtained similar propionic acid productivity (0.4 g L^−1^ h^−1^) with sugar mixtures from hydrolysis of household refuse in batch experiments. Another work (Liu *et al.*, 2012) reports lower propionic acid productivities using xylose (0.23 g L^−1^ h^−1^) and corncob molasses (0.28 g L^−1^ h^−1^) in fed-batch experiments. Dishisha *et al.* (2012) studied propionic acid production from glycerol using immobilized cells on polyethylenimine-treated Poraver (PEI-Poraver) and Luffa (PEI-Luffa). In their study, productivities of propionic acid were 0.86 and 0.29 g L^−1^ h^−1^ using PEI-Poraver and PEI-Luffa, respectively. Blanc and Goma (1987) obtained a productivity of 0.10 g L^−1^ h^−1^ for acetic acid while in the present work, the productivity of acetic acid decreased from 0.05 g L^−1^ h^−1^ in the first batch to 0.03 g L^−1^ h^−1^ in the third sequential batch (Table 4). Other results are compared in Table 5. The definitions of the abbreviations used in Table 5 can be found in Table 1.

**Table 4:** **Organic acids and cell growth productivity for the three sequential sorbitol fermentation batches using** ***P. acidipropionici*** **operating in four independent vessels**

	**First batch**	**Second batch**	**Third batch**
Succinic acid (g L^−1^ h^−1^)	0.09	0.1	0.2
Acetic acid (g L^−1^ h^−1^)	0.05	0.03	0.03
Propionic acid (g L^−1^ h^−1^)	0.6	0.5	0.5
Cell growth (g L^−1^ h^−1^)	0.06	0.09	0.14

**Table 5:** **Comparison of the results from this study with literature reports on propionic acid fermentation process by** ***P. acidipropionici*** **with different carbon sources, using free and immobilized cells in batch and fed-batches modes of operation (adapted from** (Dishisha et al. 2012) **and** Liu et al. (2012)**)**

**Strain**	**Carbon source**	**Immobilization matrix**	**Mode of operation**	**Propionic acid (g L** ^**−1**^ **)**	**Productivity (g L** ^**−1**^ **h** ^**−1**^ **)**	**Y**_**PA/S**_ **(g g** ^**−1**^ **)**	**P/A ratio (g g** ^**−1**^ **)**	**Reference**
ATCC 25562	Glycerol 30 (g L^−1^)	-	Batch	20	0.24	0.68	45.6	Barbirato et al. (1997)
ATCC 25562	Glycerol 30 (g L^−1^)	Ca alginate beads	Recycle batch	19.3	3.0	0.63	113.5	Bories et al. (2004)
ATCC 25562	Glycerol 20 (g L^−1^)	-	Batch	12	0.42	0.64	6	Himmi et al. (2000)
ATCC 4965	Glycerol 20 (g L^−1^)	-	Uncontrolled-pH Batch	6.77	0.05	0.72	Without acetic acid production	Coral et al. (2008)
CGMCC 1.2230	Glycerol 50 (g L^−1^)	-	Batch	28.53 ± 0.82	0.19	0.57	11.14 ± 0.62	Zhu et al. (2010)
	Glycerol 80 (g L^−1^)			32.00 ± 0.91	0.09	0.40	6.15 ± 0.31	
DSM 4900	Glycerol 42 ± 0.50 (g L^−1^)	-	Batch	19.46 ± 0.63	0.34	0.51	17.9	Dishisha *et al.* (2012)
	Glycerol 63.6 ± 0.90 (g L^−1^)	-		26.31 ± 0.78	0.26	0.51	47.8	
	Glycerol 43.3 ± 0.01 (g L^−1^)	PEI-Luffa		21.70 ± 0.02	0.30	0.58	27.8	
	Glycerol 63.2 ± 0.03 (g L^−1^)	PEI-Luffa		26.00 ± 0.02	0.16	0.57	38.2	
	Glycerol 42 ± 0.01 (g L^−1^)	PEI-Poraver		20.09 ± 0.01	0.86	0.51	28.7	
	Glycerol 66.6 ± 0.05 (g L^−1^)	PEI-Poraver		28.39 ± 0.02	0.43	0.51	55.7	
	Glycerol 84.6 ± 0.00 (g L^−1^)	PEI-Poraver		35.23 ± 0.01	0.35	0.47	15.3	
ATCC 4875	Xylose	-	Fed-batch	53.2	0.23	^a^	5.2	Liu *et al.* (2012)
	Corncob molasses			71.8	0.28	^a^	~4.8	
NRRLB-3569	Household refuse -	-	Batch	27.6	0.40	0.552	3.33	Blanc and Goma (1987)
ATCC 4875	Glycerol (40 g L^−1^)	-	Batch	19.3 ± 0.06	0.026 ± 0.002	0.55 ± 0.01	>100	Zhang and Yang (2009)
		Fibrous-bed bioreactor (FBB)	Fed-batch	19.7 ± 1.0	0.17 ± 0.04	0.52 ± 0.01	26 ± 4	
ATCC 4875 ACK-Tet		-	Batch	26.0 ± 0.6	0.10 ± 0.03	0.54 ± 0.02	29 ± 3	
		Fibrous-bed bioreactor (FBB)	Recycle-batch	23.0 ± 1.3	0.25 ± 0.03	0.59 ± 0.02	22 ± 2	
CGMCC1.2225 (ATCC4965)	Glycerol (40 g L^−1^)	-	Batch	18.1 ± 0.65	0.108	0.475 ± 0.017	33.5	Liu *et al.* (2011)
	Glucose (40 g L^−1^)			11.5 ± 0.45	0.068	0.303 ± 0.012	4.5	
ATCC 4875	Sorbitol (80 g L^−1^)	-	Sequential batch –first batch	39.5 ± 5.2	0.6	0.613	12.0	This work
			Sequential batch –second batch	35.8 ± 1.4	0.5	0.419	16.0	
			Sequential batch – third batch	34.4 ± 1.9	0.5	0.438	17.0	

^a^Data not calculated.

In the present study, the average propionic acid/acetic acid (P/A) molar ratio was increased from 9.7 in the first batch to 13.8 in the third sequential batch when using sorbitol as a carbon source. Liu *et al.* (2011) obtained a higher P/A molar ratio of 27.1 using glycerol and a lower P/A molar ratio of 3.63 using glucose as a carbon source. Another study (Zhu *et al.*^2010^) using glycerol as a carbon source reached a P/A mass ratio of 13.10 (P/A molar ratio of 10.6) which is similar to the P/A molar ratio of the first batch (9.7) using sorbitol as a carbon source. As expected, the propionic acid/succinic acid (P/S) molar ratio decreased from 10.3 in the first batch to 3.7 in the third sequential batch, once the concentration of succinic acid increased from 6.1 ± 2.1 to 14.8 ± 0.9 g L^−1^ and that of propionic acid decreased from 39.5 ± 5.2 to 34.4 ± 1.9 g L^−1^ (Table 6).

**Table 6:** **Propionic acid/acetic acid (P/A) and propionic acid/succinic acid (P/S) productivity ratios of three sequential sorbitol fermentation batches using** ***P. acidipropionici*** **operating in four independent vessels**

	**P/A ratio (mol mol** ^**−1**^ **)**	**P/S ratio (mol mol** ^**−1**^ **)**
First batch	9.7	10.3
Second batch	13.0	5.7
Third batch	13.8	3.7

The best propionic acid yield obtained, Y_PA/S_, was 0.613 g g^−1^ for the first batch. This result was higher than the values found in the literature; for example, Blanc and Goma (1987), reached a lower propionic acid yield of 0.552 g g^−1^ using products of hydrolysis of household refuse; Zhang and Yang (2009), using an adapted culture of *P. acidipropionici* in FBB fermentation, obtained a Y_PA/S_ value of 0.59 ± 0.02; and Liu et al. (2011), obtained a Y_PA/S_ of 0.475 ± 0.017 and 0.303 ± 0.012 from glycerol and glucose, respectively. Dishisha et al. (2012), obtained the best result for propionic acid yield (0.74 mol mol^−1^ or 0.595 g g^−1^) from glycerol. When using sorbitol, as we reported herein, it is possible to obtain a superior yield of 1.51 mol mol^−1^ (0.613 g g^−1^) (see Table 5).

Substrate consumption rates and biomass rates were similar in each batch. The succinic acid production rate increased from 0.022 g h^−1^ to 0.053 g h^−1^ while that of acetic acid (from 0.012 g h^−1^ to 0.007 g h^−1^) and propionic acid (from 0.137 g h^−1^ to 0.109 g h^−1^) decreased over the batches. The specific cell growth rate remained at a constant value of 0.014 h^−1^ over the batches (Table 7; the definitions of the abbreviations used in Table 7 can be found in Table 1). Zhang and Yang (2009) obtained a specific cell growth rate of 0.050 ± 0.002 h^−1^ when working with the original culture and 0.16 ± 0.02 h^−1^ with the adapted culture in free-cell fermentation.

**Table 7:** **Instantaneous and specific rates of the three sequential sorbitol fermentation batches using** ***P. acidipropionici*** **operating in four independent vessels**

	**First batch**	**Second batch**	**Third batch**
r_S_ = −dS/dt (g h^−1^)	−0.224	−0.272	−0.250
r_SA_ = dP/dt (g h^−1^)	0.022	0.036	0.053
r_AA_ = dP/dt (g h^−1^)	0.012	0.008	0.007
r_PA_ = dP/dt (g h^−1^)	0.137	0.114	0.109
r_x_ = dX/dt (g h^−1^)	0.082	0.089	0.070
μ_s_ = 1/X(−dS/dt) (h^−1^)	−0.039	−0.044	−0.051
μ_SA_ = 1/X(dP/dt) (h^−1^)	0.004	0.006	0.011
μ_AA_ = 1/X(dP/dt) (h^−1^)	0.002	0.001	0.001
μ_PA_ = 1/X(dP/dt) (h^−1^)	0.024	0.018	0.022
μ_x_ = 1/X(dX/dt) (h^−1^)	0.014	0.014	0.014

S: substrate (sorbitol); SA: succinic acid; AA: acetic acid; PA: propionic acid; X: cells.

In conclusion, the final concentration of propionic acid obtained in this study using sorbitol as a carbon source was higher than that obtained in other studies using glucose, household refuse, and glycerol (in some specific operation modes) presented in the literature. Employing sorbitol, an unexplored carbon source, in fermentation reactions, allowed reducing the acetic acid yield when compared to glucose and household refuse enzymatic hydrolysate as carbon sources. Furthermore, these results, all obtained in quadruplicate, are important for the development of a continuous fermentation process in the future.
